# The Impact of Hepatic Steatosis on Hepatic Ischemia-Reperfusion Injury in Experimental Studies: A Systematic Review

**DOI:** 10.1155/2013/192029

**Published:** 2013-08-24

**Authors:** Michael J. J. Chu, Anthony J. R. Hickey, Anthony R. J. Phillips, Adam S. J. R. Bartlett

**Affiliations:** ^1^Department of Surgery, University of Auckland, Private Bag 92019, Auckland 1142, New Zealand; ^2^Maurice Wilkins Centre for Biodiscovery, University of Auckland, Private Bag 92019, Auckland 1142, New Zealand; ^3^School of Biological Sciences, University of Auckland, Private Bag 92019, Auckland 1142, New Zealand; ^4^New Zealand Liver Transplant Unit, Auckland City Hospital, Private Bag 92024, Auckland 1023, New Zealand

## Abstract

*Background*. The impact of hepatic steatosis on outcome following hepatic ischemia-reperfusion injury (IRI) remains controversial with conflicting clinical results. A number of experimental studies have been published examining the relationship between hepatic steatosis and IRI. This systematic review evaluates these experimental studies. *Methods*. An electronic search of the Medline and Embase databases (January 1946 to June 2012) was performed to identify studies that reported relevant outcomes in animal models of hepatic steatosis subjected to IRI. *Results*. A total of 1314 articles were identified, of which 33 met the predefined criteria and were included in the study. There was large variation in the type of animal model, duration, and type of IRI and reporting of histological findings. Increased macrovesicular steatosis (>30%) was associated with increased histological damage, liver function derangement, and reduced survival. Increased duration of warm or cold ischemia had a negative impact on all outcomes measured. Microvesicular steatosis did not influence outcome. *Conclusions*. Findings from this systemic review support the hypothesis that livers with >30% macrovesicular steatosis are less tolerant of IRI. Clinically, it is likely that these findings are applicable to patients undergoing hepatic resection, but further studies are required to confirm these data.

## 1. Introduction

Nonalcoholic fatty liver disease (NAFLD) can present in a range of pathological states from hepatic steatosis to cirrhosis [1]. Hepatic steatosis, the early stage of NAFLD, is the most common chronic liver disease in the Western world with an estimated prevalence of 20–24% [2, 3]. As hepatic steatosis is considered the hepatic manifestation of the metabolic syndrome, its prevalence is expected to rise [4] in parallel with the increasing epidemic of obesity and the metabolic syndrome [5, 6]. The number of patients with hepatic steatosis requiring hepatic surgery is therefore likely to increase dramatically over the next decade. 

Hepatic steatosis has been associated with poor outcome following hepatic surgery [7, 8]. In orthotopic liver transplantation (OLT), moderate (>30%) and severe (>60%) steatosis of the donor organ is associated with increased rates of graft failure [7, 9, 10]. Similarly, complication rates following hepatic resection are 2-3-fold higher in patients with moderate-to-severe hepatic steatosis [8, 11]. It has been postulated that steatotic livers are less tolerant of ischemia-reperfusion injury (IRI), leading to worse clinical outcome [12, 13]. The liver is subjected to various types of IRI during hepatic surgery [14], including warm IRI in hepatic resection when hepatic inflow is temporarily occluded or cold-rewarming IRI when a donor liver is reperfused during OLT. If severe, IRI can lead to liver failure and death [15, 16]. The reason for the increased susceptibility of steatotic livers to IRI is not known. Several different hypotheses have been proposed to explain the increased susceptibility, including impaired hepatic microcirculation [17, 18] and mitochondrial dysfunction [19]. Macrovesicular steatosis is associated with accumulation of intracellular lipid, increasing hepatocyte volume leading to obstruction of the adjacent sinusoid space, and increasing the vascular resistance in the hepatic microcirculation [20, 21]. This may potentially impair oxygen and nutrient delivery following reperfusion to an already susceptible organ. The increased lipid levels in steatotic livers may also lead to mitochondrial dysfunction through the formation of reactive oxygen species [22, 23]. Mitochondrial energy supply is fundamental to cellular viability, and the interruption of key mitochondrial processes disrupts normal cellular bioenergetics, impairs cellular function, and subsequently leads to cell death by either necrosis or apoptosis [24]. Other potential mechanisms that have been proposed include Kupffer cell dysfunction [25] and impaired leukocyte adhesion [18]. It is therefore likely that the increased vulnerability of steatotic livers is multifactorial, and further experimental studies are required to elucidate the underlying mechanism. 

Currently there is no cohesive overview of the evidence relating the degree of hepatic steatosis and outcome following IRI in experimental studies. Defining this clinical relationship is imperative if one is going to potentially characterize the underlying mechanism of increased vulnerability of hepatic steatosis to IRI. The aim of this study is to systematically review the literature and describe from the available evidence the association of hepatic steatosis with outcome following hepatic IRI in experimental studies.

## 2. Methods

An electronic search was performed of the Ovid Medline and Embase databases from January 1946 to June 2012 using the following MeSH headings and keywords: [(Fat$ or steato$) and (liver or hepatic)].mp, ischemia/OR reperfusion injury/OR ischemia reperfusion.mp. The search was limited to articles published in the English language. 

The search aimed to identify all studies that reported on the outcome of animals with hepatic steatosis that were subjected to IRI. Studies were excluded if they (i) included subjects with nonalcoholic steatohepatitis rather than simple steatosis, (ii) used genetically modified animals to induce hepatic steatosis, (iii) were not original researches (systematic review, narrative review, commentary, or editorial), (iv) did not report severity and/or type of hepatic steatosis, and (v) did not report clinically relevant outcomes (graft or recipient survival, histological findings, or liver functions tests, LFT). Nonalcoholic steatohepatitis was defined as steatosis with hepatocellular injury and inflammation without fibrosis [26]. Genetically modified animals were excluded as the mutations used to induce steatosis (leptin deficiency or leptin receptor dysfunction) are not prevalent in humans, and the pathophysiology of hepatic steatosis in these animals does not mimic NAFLD in humans [27, 28]. 

Potential articles were identified using the previous search strategy. Their titles and abstracts were manually screened by the primary reviewer (M. J. J. Chu). Eligible articles were retrieved and screened in depth for eligibility and data extraction using a standardized *pro forma*. Discrepancies were adjudicated independently by the senior author (A. S. J. R. Barlett). Duplicate studies were excluded and publications with overlapping study populations, the publication with the largest number of subjects was included. Information obtained included type of animal model, severity and type of steatosis, duration and type of hepatic IRI (partial/total, warm/cold), and outcome (recipient survival, histology, or LFT). 

## 3. Results

**Figure 1:**
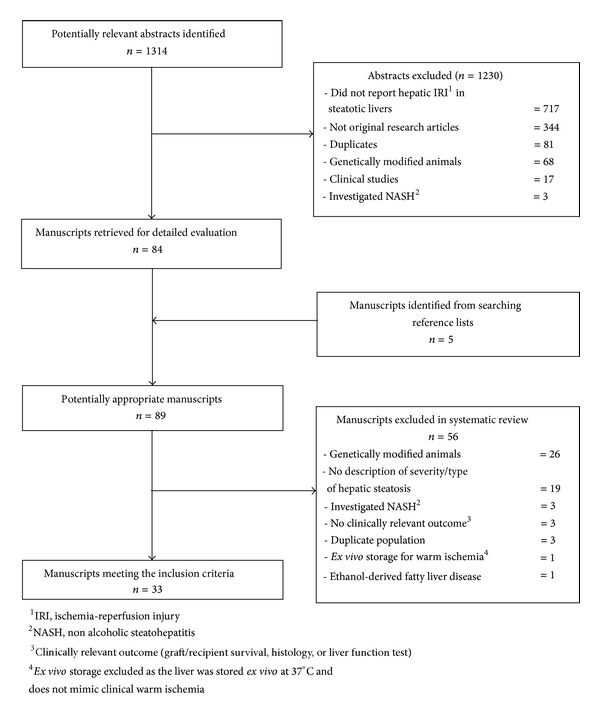
Quorum diagram.

A total of 477 and 837 articles were identified in Medline and Embase, respectively. After the exclusion of duplicates, 1233 abstracts were screened, and 84 manuscripts were obtained for further evaluation. Additional 5 manuscripts were identified from searching the reference lists. A total of 33 manuscripts fulfilled the inclusion criteria as illustrated in Figure 1 and formed the basis of this study. Among the 33 studies, 18 examined warm IRI, 14 looked at cold IRI, and 1 study investigated both warm and cold IRIs (Tables 1–8).

### 3.1. Warm IRI (Tables 1–3)

Nineteen studies examined the effect of warm IRI in hepatic steatosis. The majority (16/19) were performed on rodents, rats (*n* = 12) and mice (*n* = 4). Hepatic steatosis was induced using dietary modifications with choline-deficient diet (CDD, *n* = 8), high-cholesterol diet (HC, *n* = 3), high-fat diet (HFD, *n* = 3), dextrose with cholesterol (D-C, *n* = 2), choline-methionine-deficient diet (CMDD, *n* = 1), protein-free diet (PFD, *n* = 1), and a combination of high-fat and CMDD (*n* = 1) (Tables 1–3). A control group, receiving a standard diet, was included in 15 (79%) studies. Moderate (>30%) steatosis was present in 18 studies. Mild steatosis (<30%) was present in the remaining study. Macrovesicular steatosis was present in 14 studies, microvesicular steatosis in 2, and mixed macro- and microvesicular steatosis in 3 studies. Eight studies used partial vascular occlusion to the median and left liver lobes to induce hepatic ischemia to 70% of the liver (Tables 1–3). Six studies used total vascular occlusion while the remaining 5 studies performed partial vascular occlusion to 70% of the liver and resected the nonischemic lobes (30% of the liver) at the onset of reperfusion (Tables 1–3) [32, 34, 35, 40, 43]. The most common duration of warm ischemia was 60 minutes (*n* = 10; range 15–90 minutes). There was a wide variation in the duration of reperfusion from 30 minutes (*n* = 2), 40 minutes (*n* = 1), 60 minutes (*n* = 3), 120 minutes (*n* = 4), 180 minutes (*n* = 2), 240 minutes (*n* = 3), 360 minutes (*n* = 1), and 480 minutes (*n* = 1), 720 minutes (*n* = 1), 840 minutes (*n* = 1) to 24 hours (*n* = 7). Outcome measures included survival (*n* = 9, Table 1), histology (*n* = 12, Table 2), and LFT (*n* = 17, Table 3). 

### 3.2. Cold IRI (Tables 4–8)

Fifteen studies examined the effect of cold IRI in hepatic steatosis. The majority (14/15) were performed on rodents, rats (*n* = 12) and mice (*n* = 2). Hepatic steatosis was induced using dietary modifications with CDD (*n* = 5), HFD (*n* = 4), CMDD (*n* = 3), and a period of fasting followed by a period of fat-free diet enriched with carbohydrate (FFD-C, *n* = 3) (Tables 4–8). A control group of animals fed a standard diet was included in 11 (73%) studies. Moderate (>30%) steatosis was present in 13 studies, and 2 studies presented with mild (<30%) steatosis. Macrovesicular steatosis was present in 9 studies, and microvesicular steatosis in 1 study with the remainder having a mixed picture. Following cold ischemia, 7/15 was reperfused *in vivo* using an OLT model (Tables 4–6). The remaining 8 studies reperfused *ex vivo* using a normothermic liver perfusion circuit—isolated perfused liver model (IPM, Tables 7 and 8). The duration of cold ischemia varied from 33 minutes to 24 hours, with 24 hours being used in 4 (27%) studies. The majority of organs were flushed with University of Wisconsin (UW, University of Wisconsin, Madison, WI, USA) solution (*n* = 8), and all organs were stored for the duration of cold ischemia at 4°C on ice. Outcome measures included survival (*n* = 7, Table 4), histology (*n* = 8, Tables 5 and 7), and LFT (*n* = 12, Tables 6 and 8). 

## 4. Analysis

### 4.1. Warm IRI

All studies that reported survival following IRI (*n* = 9) demonstrated decreased survival in animals with >30% steatosis compared to lean controls [29–37]. Increased duration of ischemia [35, 36] as well as increased severity of steatosis [37] was shown to correlate with a decrease in survival. Histologically >30% macrovesicular steatosis was associated with increased intraparenchymal haemorrhage, sinusoidal congestion, and necrosis compared to lean livers [29–31, 34–41, 62]. Liver enzymes (alanine aminotransferase (ALT), aspartate aminotransferase (AST)) [29, 31, 32, 34, 35, 37–40, 42, 47, 62], prothrombin time [35], and bilirubin [35, 40] were increased in subjects with >30% macrovesicular or mixed hepatic steatosis compared to lean livers. There were 2 studies reporting on microvesicular steatosis and its impact on histological outcome and liver function [38, 39]. Both studies reported similar degree of histological injury and levels of transaminases in microvesicular steatotic animals compared to lean controls. The findings in the 5 studies that did not include a lean control were consistent with the studies of macrovesicular steatosis [30, 43–46]. Animals with lean livers (Tables 1–3) had greater survival, less histological damage, or lower liver enzymes following warm IRI compared to steatotic livers [29, 31–42, 47].

### 4.2. Cold IRI

Recipients of steatotic grafts following cold ischemia had poorer survival than those transplanted with lean livers [31, 48, 49, 52, 53]. An increased duration of cold ischemia was associated with worse recipient outcome [48, 49]. Histologically, >30% macrovesicular steatosis of the donor liver was associated with increased rate of hepatic necrosis, sinusoidal congestion, and intraparenchymal hemorrhage [31, 48, 49, 53–55]. In keeping with this, liver enzymes (ALT and AST) [31, 48, 53–55, 58, 59] and hepatic synthetic function [48, 54] were impaired compared to recipients of lean livers. However, mild (<30%) and mixed steatosis had similar levels of liver enzymes compared to lean livers following 90 minutes of cold ischemia in an IPM [57]. Similarly, severe (>60%) microvesicular steatosis of the liver was only associated with increased histological damage and deranged LFT after 24 hours of cold ischemia [56]. The findings in the 4 studies that did not include a lean control were in keeping with the results from studies of macrovesicular steatosis [50, 51, 60, 61]. Consistent with findings in warm IRI, recipients of lean livers [31, 48, 49, 52, 53] or lean livers subjected to IPM [54–61] had better outcome (survival, histological damage, and liver enzymes) following cold IRI compared to steatotic livers (Tables 4–8).

## 5. Discussion

The influence of hepatic steatosis in liver surgery is poorly described. It has been postulated that the accumulation of fat within the liver is associated with poorer patient outcome due to increased susceptibility of steatotic livers to IRI. IRI initiates a cascade of inflammation and oxidative damage that results in cellular damage [15, 16]. Inflow occlusion of the portal triad (Pringle's maneuver) [63] can be applied to decrease blood loss during liver resection, but this process of occlusion and subsequent reperfusion to the ischemic liver induces IRI that may impair liver regeneration following hepatectomy [64]. Liver transplantation is the only curative treatment for end-stage liver disease. The number of patients added to the waiting list in the United States of America from 2007 to 2009 was 10500. Over the same time period, approximately 6000 liver transplants were performed each year. This has resulted in a high mortality rate on the waiting list and forced transplant units to use marginal or extended-criteria liver grafts, which include steatotic livers [65]. In liver transplantation, the process of cold preservation and warm reperfusion leads to IRI. Although it is plausible that steatotic livers are more susceptible to IRI, it remains speculative. The prevalence of hepatic steatosis is predicted to substantially increase over the next decade in parallel with the rising prevalence of the metabolic syndrome [66]. A better understanding of the effect of hepatic steatosis in patients undergoing hepatic resection or transplantation is required, if we are going to improve the outcome of this group of patients. 

A number of experimental models of hepatic steatosis have been developed; however, a single model that encompasses the full characteristic of human NAFLD remains elusive [67]. The ideal animal model would include both the metabolic syndrome and liver pathology. Most rodent models to date have used genetically modified animals, which produce hepatic steatosis, but these mutations are not prevalent in human NAFLD pathophysiology. For this reason we did not include genetically modified models in this review. High fat and carbohydrate fed animals are probably the closest model to the clinical situation. There have only been a few reports using such models, and in this review no studies used a high fat-carbohydrate fed rodent model. The CDD or CMDD model is a compromise that has been extensively used, and was included in this review. CDD or CMDD induces hepatic steatosis through abnormal lipid metabolism but is not associated with insulin resistance, and the animals have significant weight loss. The diet used in the studies in the review that most closely resemble the clinical situation is the HFD or HC diet, and 10 studies in this review used these diets. Future studies will need to encompass the clinical dietary scenario into the experimental diet, but an ideal model remains elusive.

Hepatic steatosis is traditionally described as either macro- or microvesicular. Macrovesicular steatosis is thought to be associated with the metabolic syndrome or alcohol abuse, and microvesicular steatosis is usually related to toxins or metabolic disorders [12]. In the 33 studies identified, 23 studies reported macrovesicular steatosis of >30%. The difference in histological descriptions among the studies makes data interpretation and comparison difficult. Debate also surrounds the utility of individual staining methods or whether histological diagnosis is still the gold standard [68]. Despite this, reporting of tissue histology lacks the consistency required to define the severity of the hepatic steatosis studied as evident by the number of studies excluded due to incomplete reporting of histological description in their studies (*n* = 19). This leads to difficulty in making detailed comparisons among studies. Future studies will need to have precise identification of the type of steatosis and the percentage of steatosis involved in the experiment. This will help define a threshold for the severity of hepatic steatosis that can be correlated with adverse outcome.

In the studies included in this review, the method and duration of inducing warm IRI varied greatly. The majority of studies used partial vascular occlusion, and in a proportion of these, they resected the nonischemic lobe prior to reperfusion. This was done to force the animal to survive on the liver lobes subjected to IRI. It is unlikely that this would have influenced the outcome in these studies. In the clinical setting, total vascular occlusion is the method most commonly used in liver surgery. However, only a small number of studies (6/19) performed total vascular occlusion as this is poorly tolerated in rodents due to splanchnic congestion, with potential confounding effects from bowel ischemia and related hemodynamic disturbances. There was a large variation in the duration of ischemia and/or reperfusion between each of the studies. The most common duration of ischemia was 60 minutes, but it varied from 15 to 90 minutes, and the duration of reperfusion varied from 30 minutes to 24 hours. The duration of IRI appeared to be based on previous experience within each laboratory rather than specific evidence.

Survival after hepatectomy relies on the ability of the liver remnant to regenerate. It is postulated that steatotic livers have decreased capacity to regenerate when subjected to IRI [69]. In this review, animals with >30% macrovesicular steatosis subjected to warm IRI had a decreased rate of survival compared to nonsteatotic animals [29–37] with a clear correlation between duration of ischemia and survival. The threshold for a survivable duration of total hepatic ischemia in animals with >30% macrovesicular steatosis was 30 minutes [29, 35, 36]. All the studies utilized total hepatic ischemia to investigate survival. Apart from the duration of ischemia, the severity of steatosis also had a negative impact on survival [37]. This is consistent with the clinical suspicion that steatotic livers have a decreased ability to regenerate after hepatectomy, and the combined effect of the duration of ischemia and underlying liver disease, in this case severity of macrovesicular steatosis, should be carefully considered in the clinical setting of liver resection. 

In this review, histology and liver enzymes were used to assess severity of hepatic injury following warm IRI in 12 and 17 studies, respectively. This is similar to clinical practice where blood tests, and less commonly percutaneous biopsies, are used to monitor hepatic function. The histological findings and liver enzymes in these studies correlate with the reports of decreased survival in animals with steatotic livers. There was evidence of increased histological damage in steatotic livers compared to lean controls with corresponding greater derangements of liver function in these animals. Of note, animals with microvesicular steatosis showed similar histological findings and enzyme profile compared to lean controls [38, 39]. This is consistent with clinical studies [70, 71] where the presence of microvesicular steatosis does not influence outcome following liver transplantation. However, Llacuna et al. [72] recently reported increased histological damage and greater derangement of ALT in microvesicular steatotic animals compared to macrovesicular steatotic animals or lean controls. This raised the issue of whether the severity and type of steatosis is more important than the lipid composition of the liver in influencing the susceptibility of steatotic livers to IRI [68]. However, assessing lipid composition in a clinical setting is difficult and more invasive, but this warrants further research in humans to correlate with the experimental data.

In the studies examining the effect of cold ischemia, the duration of cold ischemia and/or reperfusion varied greatly. The duration of cold ischemia was chosen to mimic the clinical scenario of prolonged cold preservation of the donor organ. However, the duration of reperfusion is harder to standardize and varied depending on the biological factors being investigated. Another factor that is likely to affect the outcome is the model used—OLT versus IPM. The rationale for utilizing IPM is to evaluate hepatic function but in an isolated manner, removed from the influence of other physiological systems. IPM provides a controlled setting with easily reproducible experiments and absence of an immune response [73]. However, it does lack the interaction with blood components and other organ systems that the OLT model provides. Ideally, OLT should be used in experimental studies as it is an *in vivo* model with true physiological interaction, but for more targeted investigation, the IPM still provides a technically easier and cost-effective option. All of the OLT models in the included studies utilized isografts, rather than allografts. This was probably done to remove the immunological effect of alloantigens on graft outcome, focusing on the effect of steatosis. An allograft transplant model with hepatic steatosis may need to be considered for future studies to investigate whether the alloimmune response further affects the outcome of steatotic livers.

Outcome of a liver graft following transplantation is affected by the duration of cold ischemia [74], size [75], and quality [7] of the liver graft. It has been proposed that steatotic livers have decreased tolerance to prolonged cold ischemia [76] and decreased effective liver mass for transplantation [77]. The studies in this review demonstrated that >30% steatosis was associated with a lower survival rate which was further affected by the duration of cold ischemia [48, 49] and graft size [53]. Three to six hours of cold preservation of moderate macrovesicular steatotic grafts resulted in decreased survival rate and was similar to lean livers subjected to 9 hours of preservation [49]. Additionally, small lean livers (30% of standard liver volume) had good postoperative outcome whereas the same-sized steatotic liver had worse survival [53]. Furthermore, recipients of steatotic livers that were 70% of the standard liver volume had decreased survival compared to same-sized lean livers. This reaffirms the clinical suspicion that >30% macrovesicular steatosis is an independent risk factor for graft survival after transplantation, and liver grafts with >30% macrovesicular steatosis should only be transplanted if other risk factors are minimized [76]. 

## 6. Conclusions

The evidence from this systematic review suggests that livers with moderate-to-severe macrovesicular steatosis are more prone to the deleterious effects of IRI, resulting in poorer graft and recipient survival, increased histological injury, and deranged hepatic function. Due to a paucity of clinical studies looking at the influence of hepatic steatosis on patient outcome, it is unlikely that we will find the answer from a retrospective review of clinical studies, and we will need to undertake large prospective trials. Until then, clinical practice should reflect on the scientific evidence, and on the basis of the experimental evidence presented, hepatic surgeons should proceed cautiously in patients with greater than moderate macrovesicular hepatic steatosis.

## Figures and Tables

**Table 1:** Survival outcome in experimental models of hepatic warm ischemia-reperfusion injury and hepatic steatosis.

Author	Animal	Steatosis model	% steatosis	Type of steatosis	Duration of ischemia (mins, type of ischemia)	Duration of reperfusion (hours)	Survival of steatotic livers (lean livers)
Ellett et al. [29]	Mouse	HFD	30–60	MaS	35 (total)	24 hours	31% (85%)^1^
Yamagami et al. [30]	Rat^2^	CDD	40–60	MaS	45 (total)	7 days	33.3%
He et al. [31]	Mouse	HFD	50–60	MaS	45 (total)	24 hours	33% (100%)^1^
Caraceni et al. [32]	Rat	CDD	50–60	MaS	60 (total)	7 days	60% (100%)^1^
Selzner et al. [33]	Mouse	CDD	>60	Mixed	60 (total)	14 days	80% (100%)^1^
Caraceni et al. [34]	Rat	CDD	>60	MaS	60 (total)	7 days	64% (100%)^1^
Hakamada et al. [35]	Rat	CDD	>60	MaS	30, 60, or 90 (total)	7 days	95%, 10%, and 5% (100%, 90%, and 35%)^1^
Hui et al. [36]	Rat	CDD	>70	MaS	30, 45, or 60 (total)	7 days	75%, 20%, and 0 (100%, 90%, and 70%)^1^
Takahashi et al. [37]	Canine	HFD	7–99	MaS	60 (total)	24 hours	100% in <30% MaS 0 in >30% MaS (100%)^1^

CDD: choline-deficient diet; HFD: high-fat diet; MaS: macrovesicular steatosis; Mixed: presence of both macrovesicular and microvesicular steatosis; ^1^ *P* < 0.05 versus lean livers; ^2^no lean group in study.

**Table 2:** Histological findings in experimental models of hepatic warm ischemia-reperfusion injury and hepatic steatosis.

Author	Animal	Steatosis model	% steatosis	Type of steatosis	Duration of ischemia (mins, type)	Duration of reperfusion (mins)	Outcome measures	Results in steatotic livers (lean livers)
Yamada et al. [38]	Rat	D-C	30–60	MiS	30 (partial)	240	HIS	5 (5)^1^
Yamada et al. [39]	Rat	D-C	30–60	MiS	30 (partial)	24 hours	HIS	6 (6)^1^
Ellett et al. [29]	Mouse	HFD	30–60	MaS	35 (total)	24 hours	HIS	1.4 ± 0.1 (0.4 ± 0.1)^2^
Yamagami et al. [30]	Rat^3^	CDD	40–60	MaS	45 (total)	40 or 180	Histo	Severe congestion and necrosis
He et al. [31]	Mouse	HFD	50–60	MaS	45 (total)	24 hours	HIS	1.8 ± 0.2 (0.9 ± 0.1)^2^
Marsman et al. [40]	Rat	CMDD	>60	MaS	40 (total)	24 hours	Histo (% necrosis)	37 ± 10 (5 ± 1%)^2^
Andraus et al. [41]	Rat	PFD	>60	MaS	60 (partial)	240	Histo	↑ Intraparenchymal haemorrhage
Selzner et al. [33]	Mouse	CDD	>60	Mixed	45 or 60 (partial)	24 hours	Histo (% necrosis)	65 ± 24% (25 ± 12%)^2^
Caraceni et al. [34]	Rat	CDD	>60	MaS	60	30 to 24 hours	Histo	↑ Sinusoidal congestion and necrosis
Hakamada et al. [35]	Rat	CDD	>60	MaS	30 or 60 (total)	360	Histo	↑ Sinusoidal congestion and necrosis
Hui et al. [36]	Rat	CDD	>70	MaS	30, 45, or 60	60	Histo	Severe necrosis and haemorrhage (no morphological change)
Takahashi et al. [37]	Canine	HFD	7–99	MaS	60	24 hours	Histo	↑ Sinusoidal congestion and necrosis

CDD: choline-deficient diet; CMDD: choline-methionine-deficient diet; D-C: dextrose with cholesterol; HFD: high-fat diet; HIS: histological injury score; Histo: histology; MaS: macrovesicular steatosis; MiS: microvesicular steatosis; Mixed: presence of both macrovesicular and microvesicular steatosis; PFD: protein-free diet.

^1^No significant difference between steatotic and lean livers; ^2^ *P *< 0.05 versus lean livers; ^3^no lean group in study.

**Table 3:** Liver function tests in experimental models of hepatic warm ischemia-reperfusion injury and hepatic steatosis.

Author	Animal	Steatosis model	% steatosis	Type of steatosis	Duration of ischemia (mins, type)	Duration of reperfusion (mins)	Outcome measures	Results in steatotic livers (lean livers)
Luo et al. [42]	Mouse	HFD + CMDD	<30	Mixed	15 (total)	180	ALT AST	450 ± 100 (120 ± 10 IU/L)^1^ 480 ± 100 (210 ± 20 IU/L)^1^

Domenicali et al. [43]	Rat^2^	CDD	>30	MaS	60 (total)	120	ALT	7500 ± 500 IU/L

Yamada et al. [38]	Rat	D-C	30–60	MiS	30 (partial)	240	ALT	4590 ± 811 (4951 ± 788 IU/L)^3^

Yamada et al. [39]	Rat	D-C	30–60	MiS	30 (partial)	24 hours	ALT	476 ± 244 (368 ± 97 IU/L)^3^

Ellett et al. [29]	Mouse	HFD	30–60	MaS	35 (total)	24 hours	ALT	290 ± 120 (130 ± 30 IU/L)

Koti et al. [44]	Rat^2^	HC	30–60	MaS	45 (partial)	120	ALT AST	5436.3 ± 984.7 IU/L 3166.3 ± 379.6 IU/L

Yamagami et al. [30]	Rat^2^	CDD	40–60	MaS	45 (total)	40 or 180	ALT AST	518.7 ± 80.3 IU/L 467.7 ± 185 IU/L

He et al. [31]	Mouse	HFD	50–60	MaS	45 (total)	24 hours	ALT	180 ± 10 (80 ± 5 IU/L)^1^

Fusai et al. [45]	Rabbit^2^	HC	30–60	MaS	60 (partial)	360	ALT	280 ± 10 U/mL

Hafez et al. [46]	Rabbit^2^	HC	30–60	MaS	60 (partial)	420	ALT AST	178 ± 34 IU/L 406 ± 86 IU/L

Caraceni et al. [32]	Rat	CDD	50–60	MaS	60 (total)	120	ALT	4000 ± 300 IU/L (1000 ± 50 IU/L)^1^

Marsman et al. [40]	Rat	CMDD	>60	MaS	40 (total)	24 hours	ALT AST Bili	1500 ± 200 (500 ± 100 IU/L)^1^ ↑ AST (Data not shown)^1^ 14 ± 3 (6 ± 1 *μ*mol/L)^1^

Selzner et al. [33]	Mouse	CDD	>60	Mixed	45 or 60 (partial)	24 hours	AST	16566 ± 5731 (500 ± 3395 IU/L)^1^

Caraceni et al. [34]	Rat	CDD	>60	MaS	60	30 to 24 hours	ALT	8106 ± 1125 (3872 ± 400 IU/L)^1^

Hakamada et al. [35]	Rat	CDD	>60	MaS	30 or 60 (total)	360	ALT Bili PT	1851.4 ± 66.2 (1861.5 ± 212.6 IU/L)^4^ 0.67 ± 0.1 (0.14 ± 0.02)^1^ 21.0 ± 0.9 (18.4 ± 1.3)^4^

Rolo et al. [47]	Rat	CDD	>60	Mixed	90 (partial)	12 hours	ALT AST	1820 ± 5 (1450 ± 20 IU/L)^1^ 1790 ± 100 (1190 ± 100 IU/L)^1^

Takahashi et al. [37]	Canine	HFD	7–99	MaS	60	24 hours	ALT	1400 ± 50 (600 ± 50 IU/L)^1^

ALT: alanine aminotransferase; AST: aspartate aminotransferase; Bili: bilirubin; CDD: choline-deficient diet; CMDD: choline-methionine-deficient diet; D-C: dextrose with cholesterol; HC: high cholesterol; HFD: high-fat diet; MaS: macrovesicular steatosis; MiS: microvesicular steatosis; Mixed: presence of both macrovesicular and microvesicular steatosis; PT: prothrombin time.

^1^ *P *< 0.05 versus lean livers; ^2^no lean group in study; ^3^no significant difference between steatotic and lean livers; ^4^ALT and PT were significantly higher in steatotic livers compared to lean livers after 30 minutes of ischemia but not after 60 minutes of ischemia.

**Table 4:** Survival outcome in experimental models of orthotopic liver transplantation and hepatic steatosis.

Author	Animal	Steatosis model	% steatosis	Type of steatosis	Duration of ischemia (mins)	Perfusate	Duration of reperfusion	Survival of recipients of steatotic livers (lean livers)
Astarcioglu et al. [48]	Rat	CDD	30–60	MaS	60 or 540	ns	7 days	87.5 and 0 (100 and 100%)^1^
Hayashi et al. [49]	Rat	CDD	30–60	MaS	60 to 540	UW	Up to 7 days	0 (75%)^1^
Cheng et al. [50]	Rat^2^	CMDD	<30 to >30	Mixed	33–39	UW	ns	33.3% (100%)^1,3^
He et al. [31]	Mouse	HFD	50–60	MaS	180	Saline	7 days	0 (40%)^1^
Schmeding et al. [51]	Rat^2^	CDD	>50	MaS	720	UW	Up to 7 days	68%
Berthiaume et al. [52]	Rat	CMDD	>60	MaS	720	UW	Up to 7 days	0 (85%)^1^
Morioka et al. [53]	Rat	HFD	40–50	Mixed	120	HTK	7 days	↓ Survival^4^

CDD: choline-deficient diet; CMDD: choline-methionine-deficient diet; HFD: high-fat diet; HTK: histidine-tryptophan-ketoglutarate solution; MaS: macrovesicular steatosis; Mixed: presence of both macrovesicular and microvesicular steatosis; ns: not stated; Saline: normal saline solution; UW: University of Wisconsin solution.

^1^ *P* < 0.05 versus lean livers; ^2^no lean group in study; ^3^decreased survival in >60% macrovesicular steatosis compared to <60% macrovesicular, >60% microvesicular steatosis or >60% mixed steatosis; ^4^decreased survival in recipients of 30 and 70% steatotic liver volume compared to recipients of volume matched lean livers.

**Table 5:** Histological finding in experimental models of orthotopic liver transplantation and hepatic steatosis.

Author	Animal	Steatosis model	% steatosis	Type of steatosis	Duration of ischemia (mins)	Perfusate	Duration of reperfusion (mins)	Outcome measures	Results in recipients of steatotic livers (lean livers)
Astarcioglu et al. [48]	Rat	CDD	30–60	MaS	60 or 540	ns	120	Histo	↑ Parenchymal injury
Hayashi et al. [49]	Rat	CDD	30–60	MaS	60 to 540	UW	Up to 7 days	Histo	↑ Sinusoidal congestion and necrosis
He et al. [31]	Mouse	HFD	50–60	MaS	180	Saline	24 hours	HIS	2.4 ± 0.05 (1.8 ± 0.05)^1^
Schmeding et al. [51]	Rat^2^	CDD	>50	MaS	720	UW	Up to 7 days	Histo	Severe necrosis
Morioka et al. [53]	Rat	HFD	40–50	Mixed	120	HTK	48 hours	Histo	↑ Sinusoidal congestion and necrosis

CDD: choline-deficient diet; HFD: high-fat diet; Histo: histology; HIS: histological injury score; HTK: histidine-tryptophan-ketoglutarate solution; MaS: macrovesicular steatosis; Mixed: presence of both macrovesicular and microvesicular steatosis; ns: not stated; Saline: normal saline solution; UW: University of Wisconsin solution.

^1^ *P *< 0.05 versus lean livers; ^2^no lean group in study.

**Table 6:** Liver function tests in experimental models of orthotopic liver transplantation and hepatic steatosis.

Author	Animal	Steatosis model	% steatosis	Type of steatosis	Duration of ischemia (mins)	Perfusate	Duration of reperfusion (mins)	Outcome measures	Results in recipients of steatotic livers (lean livers)
Astarcioglu et al. [48]	Rat	CDD	30–60	MaS	60 or 540	ns	120	ALT AST Bile production	1640 ± 482 (396 ± 54 IU/L)^1^ 2270 ± 684 (580 ± 70 IU/L)^1^ 6.2 ± 0.5 (23.8 ± 1.8 cm/10 min)^1^
He et al. [31]	Mouse	HFD	50–60	MaS	180	Saline	24 hours	ALT	10000 ± 1500 (5000 ± 200 IU/L)^1^
Schmeding et al. [51]	Rat^2^	CDD	>50	MaS	720	UW	Up to 7 days	ALT AST	1200 ± 900 IU/L 1500 ± 1200 IU/L
Morioka et al. [53]	Rat	HFD	40–50	Mixed	120	HTK	48 hours	ALT	700 (200 IU/L)^1^

ALT: alanine aminotransferase; AST: aspartate aminotransferase; CDD: choline-deficient diet; HFD: high-fat diet; HTK: histidine-tryptophan-ketoglutarate solution; MaS: macrovesicular steatosis; Mixed: presence of both macrovesicular and microvesicular steatosis; ns: not stated; Saline: normal saline solution; UW: University of Wisconsin solution.

^1^ *P* < 0.05 versus lean livers; ^2^no lean group in study.

**Table 7:** Histological finding in experimental models of isolated perfused model and hepatic steatosis.

Author	Animal	Steatosis model	% steatosis	Type of steatosis	Duration of ischemia (mins)	Perfusate	Duration of reperfusion (mins)	Outcome measures	Effect of hepatic steatosis
von Heesen et al. [54]	Rat	FFD-C	40–50	MaS	24 hours	HTK	60	Histo	↑ Necrosis
Baskin-Bey et al. [55]	Mouse	CMDD	>40	MaS	24 hours	UW	60	Histo	↑ Liver injury
Arnault et al. [56]	Rat	HFD	80–100	MiS	12, 18, or 24 hours	UW	180	Histo	↑ Haemorrhage^1^

CMDD: choline-methionine-deficient diet; HFD: high-fat diet; FFD-C: fat-free diet enriched with carbohydrate; Histo: histology; HTK: histidine-tryptophan-ketoglutarate solution; MaS: macrovesicular steatosis; MiS: microvesicular steatosis; UW: University of Wisconsin solution.

^1^Only in livers preserved for 24 hours.

**Table 8:** Liver function tests in experimental models of isolated perfused model and hepatic steatosis.

Author	Animal	Steatosis model	% steatosis	Type of steatosis	Duration of ischemia (mins)	Perfusate	Duration of reperfusion (mins)	Outcome measures	Results in steatotic livers (lean livers)
Jamieson et al. [57]	Porcine	HFD	28	Mixed	90	Soltran	48 hours	ALT/AST	Similar ALT/AST
von Heesen et al. [54]	Rat	FFD-C	40–50	MaS	24 hours	HTK	60	ALT AST Bile production	400 ± 100 (20 ± 5 IU/L)^1^ 250 ± 60 (15 ± 5 IU/L)^1^ 0 (0.16 ± 0.05 *μ*L/g/min)^1^
Baskin-Bey et al. [55]	Mouse	CMDD	>40	MaS	24 hours	UW	60	ALT	↑ ALT^1^ (levels not specified)
Caraceni et al. [58]	Rat	CDD	50–60	MaS	18 hours	UW	120	ALT	664 ± 77 (140 ± 30 IU/L)^1^
Caraceni et al. [59]	Rat	CDD	50–60	MaS	18 hours	UW	120	ALT	64.9 ± 10.7 (6.4 ± 1.7 IU/L)^1^
Puetz et al. [60]	Rat	FFD-C	<60	Mixed	ns	HTK	45	ALT	↑ ALT^1^ (levels not specified)
Minor et al. [61]	Rat	FFD-C	<60	Mixed	24 hours	HTK	45	ALT	434.3 ± 70.2 (2.5 ± 0.2 IU/L)^1^
Arnault et al. [56]	Rat	HFD	80–100	MiS	12, 18, or 24 hours	UW	180	ALT/AST Bile production	↑ ALT/AST^2^ ↓ Bile production^2^

ALT: alanine aminotransferase; AST: aspartate aminotransferase; CDD: choline-deficient diet; CMDD: choline-methionine-deficient diet; HFD: high-fat diet; FFD-C: fat-free diet enriched with carbohydrate; HTK: histidine-tryptophan-ketoglutarate solution; MaS: macrovesicular steatosis; MiS: microvesicular steatosis; Mixed: presence of both macrovesicular and microvesicular steatosis; ns: not stated; Soltran: Marshall's hypertonic citrate; UW: University of Wisconsin solution.

^1^ *P* < 0.05 versus lean livers; ^2^only in livers preserved for 24 hours.
